# Tracing Sources and Contamination Assessments of Heavy Metals in Road and Foliar Dusts in a Typical Mining City, China

**DOI:** 10.1371/journal.pone.0168528

**Published:** 2016-12-16

**Authors:** Jie Yang, Yanguo Teng, Liuting Song, Rui Zuo

**Affiliations:** 1 College of Water Sciences, Beijing Normal University, Beijing, China; 2 Engineering Research Center of Groundwater Pollution Control and Remediation, Ministry of Education, Beijing, China; Boston University, UNITED STATES

## Abstract

Road and foliar dust samples from four land-use districts of Panzhihua City, a famous V-Ti magnetite production area of China, were collected to investigate the sources and distribution characteristics of 9 heavy metals (V, Pb, Cd, Cu, Zn, Ni, Cr, Fe, and Mn). The results suggest that foliar samples had smaller particle size and higher heavy metal contents than road dusts. The contamination assessments of heavy metals were as follows: Pb and V (significant enrichment) > Zn, Ni, Cr, Fe, and Mn (moderate enrichment) > Cd and Ni (minimal enrichment). Statistical analyses showed Pb, as the primary pollution element, originated from waste incineration and lead-fuel combustion. The sources of Zn, Ni, Cr, Fe, V, and Mn were fugitive dust and traffic activities. Potential origins of Cu were corrosion of alloys used in vehicle components, vehicle covers, or other metallic surfaces and materials. The sources of Cd were different from any other heavy metals. Traffic and industrial activities were the main anthropogenic origins of heavy metals in dusts of Panzhihua, and more attention should be paid to heavy metal pollution in agricultural area.

## Introduction

The urban environment is strongly influenced by human activities including the growth of population, industrial activities and vehicles in large cities. Such human activities result in the significant enrichment of heavy metals in soil, on roads, and on building and foliage surfaces [1–5]. Road dust is the particles from atmospheric dust and other urban non-point sources deposited on the impervious pavement of the street by wind, hydraulics and gravity, which have become one of the most widespread pollutant carriers on the surface [6,7]. In addition, foliar dust can be formed by the retention of atmospheric particles on the special surface organization of plant [5]. Through long-term accumulation, the components of foliar dust are more complex and can show the input characterization of elements at a certain time and region, which is a good indication of the environmental conditions [5].

Heavy metals in road and foliar dust may originate from anthropogenic sources such as steel-iron refining, electronics and dyeing, diesel and coal combustion, waste incineration, etc. In highly populated cities, heavy metals in road and foliar dust are potential contaminants with direct or indirect contact with the skin, hands, or mouth [8, 9]. Heavy metals are not biodegradable and can remain in dust over long periods of time. A few metals (Cu, Zn, Fe and Mn) have little effect on humans, while others (Pb, Cd, and Cr)have serious effects even at low concentrations [10]. During rain/storm/street-washing events, dust particles laden with contaminants are washed off and finally end up in receiving water bodies[11, 12].

Road dust contamination has received great attention in recent years [6, 13–17]. Lu [15] based on enrichment factor analysis, conducted a contamination assessment of Cu, Pb, Zn, Mn and Ni in street dusts of Baoji. Such multivariate analyses have been widely used to identify probable sources and ecological risk assessments of heavy metals in road dust [16, 18]. Furthermore, some researchers made further discussions to compare the metal contents of road dust in typical land-use districts. Gunawardana et al. analyzed the mineralogy and morphology of road dusts in four cities of Australia, and land use of each of the sampling sites was divided into residential, industrial, and commercial areas [13]. Acosta [6] compared the influence of population density on the concentration and speciation of metals in road dust.

Compared with road dust, foliar dust is accumulated over a longer time (the urban street will be cleaned regularly) and is a mixture of atmospheric particulate matter and resuspended dust. Foliar dust is a necessary way for the study of urban pollutants while there is less relative research about it. The dust-retention abilities of plants depend on several factors, such as the different types of tree canopy, branch and leaf density, leaf morphology, and prevailing meteorological conditions [7, 19, 20]. Qiu [7] found that the concentrations of Cd and Pb in foliar dusts were particularly high in Huizhou, China. In Vienna, Austria, elemental concentrations of foliar dusts were significantly higher in urban area than in rural area [21].

Urbanization and industrialization in Panzhihua has taken place at an unprecedented pace in the last three decades, which had caused high pollution pressure on the local environment. In this paper, 9 heavy metals (V, Pb, Cd, Cu, Zn, Ni, Cr, Fe, and Mn) were investigated in road and foliar dusts of different land-use districts of Panzhihua. The major objectives were to assess the distribution and pollution levels of the metals, and identify potential sources of these elements.

## Materials and Methods

### Study area

Panzhihua City, which is between eastern longitudes 101°15′ to 102°08′ and northern latitudes 26°05′ to 27°21′, is located in Sichuan Province, southwest China, and beside the Yalong River and Jinsha River. As the southern part of NS-trending Panxi rift valley, Panzhihua has complex geological structure. The main exposed strata are Proterozoic, Paleozoic and Mesozoic, and magmatic rock is most developed. Its terrain is sloping from northwest to southeast and is divided into three major areas and two canyons by Yalong River and Jinsha River. The western region of Panzhihua is an important tectonic metal logenic belt in China and 76 kinds of mine have been found in this district. Among them, the giant Panzhihua Vanadium-Titanium Magnetite [Fe (V, Ti)_3_O_4_] deposit, which is celebrated as the most famous base of V-Ti magnetite production in the world, provides 20% Fe, 64% V and 53% Ti supply for China.

This district has a variety of subtropical and temperate climate types with large differences in temperature between day and night, and the average temperature is in the range of 19.7°C to 20.5°C with long sunshine (2300–2700 hr/a) and strong solar radiation (578–628 KJ/cm^2^). Panzhihua City is governed by three districts (East District, West District, and Renhe District) and two counties (Miyi County and Yanbian County). The East District is the main urban area of Panzhihua City, which is the city's political, economic, cultural, financial and business center.

### Sample collection and preparation

After making a detailed investigation of the cultural, industrial, commercial and residential distribution of Panzhihua City, part of the Sutie East Road, Jinsha Jiang Road, and Panzhihua road (approximately 17 km long), which connects the East and West Districts of Panzhihua City, were chosen as the sampling area. All of sampling sites were along the roads, and the roads accessed were owned by the state and not privately owned or protected. The paper was granted by Ministry of environmental protection of the People's Republic of China, and relative introduction of study area was obtained from the Panzhihua public information network. The field studies did not involve endangered or protected species.

**Fig 1:**
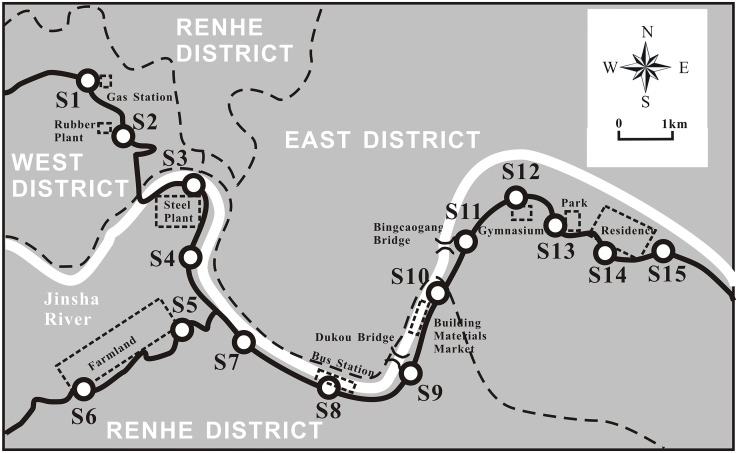
Foliar and road dust samples collection sites.

As shown in Fig 1, a total of 15 sampling points with an interval of 1-2km were selected in this area including the Industrial Area (IA) (S01, S02, S03), Agricultural Area (AA) (S04, S05, S06, S07), Heavy Traffic Area (HTA) (S8, S9, S10, S11), and Residential Area(RA) (S12, S13, S14, S15). There were one gas station, one rubber plant and one steel plant in IA, the vicinity was easily influenced by industrial activities, such as vehicle repair, vehicle refueling, rubber sulphuration, rubber mastication, and steel rolling process. The site of S04 and S07 of AA were vegetable filed, while S05 and S06 were in hillside orchard. Though they were divided into AA, they were still influenced by traffic and irrigation. The HTA was located among the Dukou and Bingcaogang Bridge where the most important flyovers had in east district. The roads in this district had two more lanes than other districts. An inter-city bus station and building materials market were also in this area. So the heavy traffic was the main human activities in HTA. The sites (S12-S15) of RA were distributed in city center which had the highest population density.

Rainfall is centralized from June to October in Panzhihua City, and road and foliar dust samples were collected on a calm and cloudless day in late November 2012. To avoid the influence of rush hour traffic and city cleaning time, 15:00–16:00 in the afternoon was selected as the sampling time.

At each sampling site (Fig 1), approximately 200g of road dust was sampled within an area of approximately 2m×2m from the edge of the road using a new broom and plastic spatula and stored in polyethylene bags. Before testing, all of the road dust samples were dried in air, sieved through d < 0.5 mm, and then stored in polyethylene bags.

Loquat (*Eriobotrya japonica*, Thunb. Lindl.) was the main tree species in green belts of Panzhihua, which had glazed surface leaves. Around each sampling site, loquat leaves were collected to obtain foliar dust samples. To avoid the errors caused by sampling, leaves of similar size and height (1.4m-1.6m) were selected and the sampling location included the east, west, south, and north parts of the branches. Polyethylene gloves were used during sampling, and leaf samples were stored in polyethylene bags. The foliar dust on the leaf surfaces at each site was brushed three times into a separate beaker with pure water (T. D. S.≤0.50ms·cm-1, 25°C). Each eluent was transferred to one 500-ml beaker and heated on a furnace. When only 10ml of the solution was left, the sample was transferred to an evaporating basin and dried with a water bath heated to 95°C. The solids that were left were foliar dusts and stored in polyethylene bags after being sieved at d < 0.5 mm. The particle size of the dust samples of Panzhihua was detected by a laser particle sizer (Rise200, Jinan, China), and the measurements ranged from 0.1 to 600μm (accuracy error <± 3%).

### Chemical analyses

Total metal contents (V, Pb, Cd, Cu, Zn, Ni, Cr, Fe, and Mn) of road and foliar dust samples were determined after aqua regia digestion following the method of ISO 11466 [22]. In this case, the amount of acid used to digest 1 g of the sample was reduced to keep the same volume/mass ratio: 7.0 ml of HCl (37%) and 2.3 ml of HNO_3_ (70%) were added. The total content were measured by inductively coupled plasma atomic emission spectrometry (ICP-AES, ULTIMA, France), and the detection limit of each elements was 0.001 (V), 0.004 (Pb), 0.0002 (Cd), 0.001 (Zn), 0.001 (Ni), 0.001 (Cr), 0.001 (Fe), 0.0001 (Mn) μg/ml. To ensure the accuracy of the results, the tests were repeated for three times for every sample. The relative standard deviations of each sample were less than 4.81%. So the average value of three test results was used in following discussion. The standard reference material (ESS-3) of road dust was obtained from the China National Environmental Monitoring Center was selected to control the data quality. The recoveries for all of the elements were greater than 95% of the certified values accompanied by the standard reference material. All of the chemicals used in the experiment were of analytical grade, and deionized water was used for all of the procedures.

### Methods of sources identification

#### Cluster analysis

Cluster analysis is the process of grouping similar objects together from diversified data, which is wildly used to identify the potential sources of contaminations. An R-type Cluster analysis based on the Pearson correlation method was chosen to show the similarities between the variables (metal concentrations. Through the SPSS Statistics 20, the between-groups linkage method with squared euclidean distance was used between two samples and the highest similarities were clustered first. The results of hierarchical cluster analysis were shown in dendrograms.

#### Enrichment factors

Enrichment factors (EFs) are widely used to analyze the enrichment of metals in dusts and to determine whether they were from natural or anthropogenic sources. EFs can be calculated according to the following:

$$EF=\frac{{\left(C_{x}/C_{ref}\right)}_{sample}}{{\left(C_{x}/C_{ref}\right)}_{background}}$$ (1)

Where (C_x_/C_ref_) is the ratio of the concentrations of heavy metal to the concentration of a reference metal in study samples and background value. The reference metal is selected based on statistical data that show no or minimal effects on human activities and are stable and widely exist in dusts [15, 16]. Five contamination categories are recognized on the basis of the EFs: EF<2, minimal enrichment; 2≤EF<5, moderate enrichment; 5≤EF<20, significant enrichment; 20≤EF<40, very high enrichment; and EF≥40, extremely high enrichment [15, 23].

#### Principal component analysis

Principal component analysis is a multivariate statistical method, which is based on the relationship between multiple variables and use key factors to explain the complex information. The factor analysis was firstly carried on to calculate the eigenvalues and characteristic variable of matrix. After determining the common factor number and initial load factor, the initial factor load was matrix to get target factor load. And finally calculate the cumulative percent of target factor load and factor variance. When the cumulative percent was greater than 90%, the principal component extracted from the variables can represent the main source information. To reduce the number of variables, a varimax rotation method was used to produce one rotated component matrix, and principal factors >0.6 were underlined in each column. The principal component analysis process was calculated through SPSS statistics 20.

## Results and Discussion

### Particle size distribution

**Fig 2:**
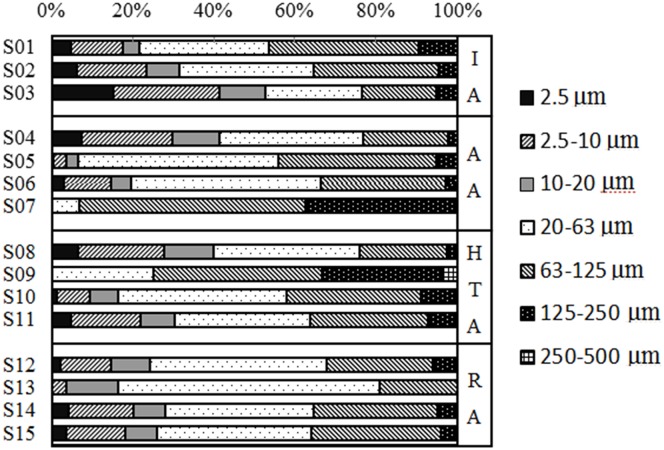
The percentage of road dusts particle size. The samples including four districts: (IA) Industrial Area; (AA) Agricultural Area; (HTA) Heavy Traffic Area; (RA) Residential Area.

**Fig 3:**
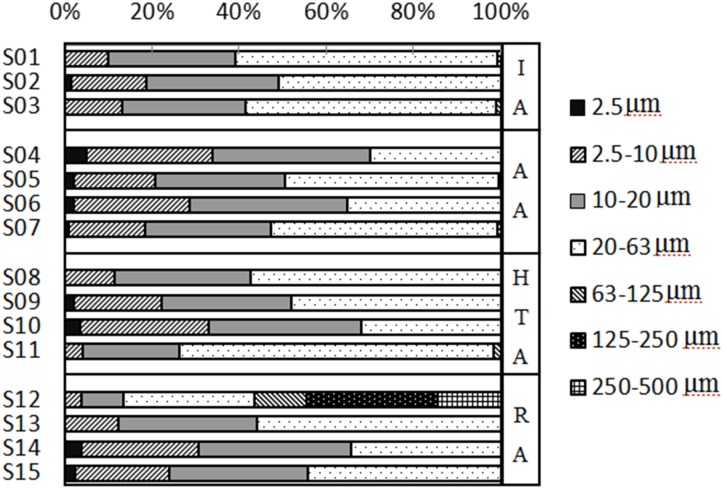
The percentage of foliar dusts particle size. The samples including four districts: (IA) Industrial Area; (AA) Agricultural Area; (HTA) Heavy Traffic Area; (RA) Residential Area.

The grain size distribution of dust is particularly important because it determines the mobility and sources of the particles [24, 25]. To illustrate the characteristics of foliar and road dust particle size from Panzhihua City, the particles were placed in classes according to the following fractions: ≤2.5μm (clay), 2.5–10μm (silt), 10–20μm (silt), 20–63μm (silt), 63–125μm (very fine sand), 125–250μm (find sand), and 250–500 μm (medium sand) as shown in Figs 2 and 3. The specific area and size distribution parameters (D_10_, D_50_, D_90_, and D_av_) of the particles were also analyzed (Table 1).

**Table 1:** Distribution of particle sizes of foliar (FD) and road dusts (RD) in Panzhihua city (N = 15).

Study Area	Sampling point	D_10_ (μm)		D_50_ (μm)		D_90_ (μm)		D_av_ (μm)		Specific area (m^2^·cm^-3^)	
		FD	RD	FD	RD	FD	RD	FD	RD	FD	RD
IA	S01	10.02	4.23	23.24	60.29	44.31	126.32	21.26	36.60	0.38	0.49
	S02	6.92	3.49	20.01	45.39	39.16	108.24	16.53	25.09	0.53	0.61
	S03	8.89	1.96	22.76	16.60	45.48	104.67	20.59	14.44	0.41	1.32
AA	S04	3.73	3.02	15.12	28.57	36.03	92.65	12.42	19.77	0.80	0.75
	S05	6.28	24.98	19.53	59.53	41.24	109.43	15.80	49.57	0.56	0.20
	S06	5.31	5.91	15.27	66.72	31.70	141.29	12.98	40.27	0.67	0.40
	S07	7.16	69.73	20.59	114.35	42.57	172.59	17.14	109.83	0.50	0.07
HTA	S08	9.46	3.30	21.93	29.74	40.38	94.60	19.95	19.73	0.40	0.70
	S09	5.96	44.39	18.95	98.77	39.07	200.09	15.58	94.72	0.59	0.09
	S10	4.67	10.43	14.10	55.54	31.20	124.60	12.10	37.08	0.74	0.30
	S11	13.11	4.02	28.07	45.62	50.03	117.93	25.96	26.14	0.30	0.56
RA	S12	16.54	6.57	102.39	43.88	290.78	111.09	73.74	30.72	0.17	0.41
	S13	9.18	15.35	21.52	37.86	40.66	81.10	19.72	34.42	0.41	0.24
	S14	4.52	4.40	14.88	46.84	31.71	109.40	12.33	27.52	0.74	0.53
	S15	5.65	5.07	17.79	48.70	36.91	106.39	14.52	27.87	0.62	0.48

D_10_, D_50_, D_90_: the particle size of dusts reach the value of 10%, 50%, and 90%on the particle size distribution curve. D_av_: the average size of particle.

For foliar dusts, 93.33% of samples were smaller than 63 μm, and their median and average particle sizes (D_50_ and D_av_) were in the range of 14%-28.07% and 12.10%-25.96% (Fig 3). Thus, most particles in foliar dusts belonged to silt and clay. Only samples of S12 had 56.1% of the particles greater than 63 μm including 11.93% of very fine sand, 29.82% of fine sand, and 14.35% of medium sand. Therefore, the sources of S12 foliar dust may be different from others. As shown in Fig 2, the particles of <250 μm represented the largest fraction of road dusts, especially in the range of 20 to 125 μm. The S7 road dust sample was generally enriched in very fine and fine sand, while the others consisted of silt, very fine and fine sand. Overall, the grain sizes of foliar dust were smaller than road dust, which was the same as the informed research [5, 26]. For the difference of location deposition height, the sources of road dusts were more complex than foliar dusts. The distribution of leaf dust partials were influenced by the plant species and environmental conditions, which were related to the source of dusts and the microstructure of the leaves [21]. Smaller particles could stay in the air for a longer time, and transported long distance with the changes of meteorological conditions. So the fine particles might come from far away in green space [26]. Metals in the fine fraction were generally considered to arise from exhaust emissions, whereas metals in the coarse particles were considered to be derived from the components of wear and tear of vehicles [27, 28].

Particle size fractions with a diameter smaller than 2.5μm (PM_2.5_) and 10μm (PM_10_) in atmosphere are the most potentially dangerous pollution for humans. PM_10_ is usually deposited in the upper respiratory tract, while PM_2.5_ can go further to the bronchioles and alveoles [29, 30]. A portion of PM_10_ and PM_2.5_ will deposit on the road, buildings or foliage and may be transported back to the atmosphere or water bodies, alveoles under special conditions [31]. In different land-use districts, the percentages of particles smaller than 2.5μm in road and foliar dusts were 5.06–15.25% and 0–1.39% in IA, 0–7.45% and 1–5.02% in AA, 0–6.54% and 0–3.39% in HTA, and 0–4.48% and 0–3.7% in RA, respectively. The percentages of particles between 2.5μm and 10μm in road and foliar dusts were 12.68–26.23% and 9.8–17.33% in IA, 3.01–22.38% and 18.82–28.92% in AA, 0–21.26% and 4.08–29.47% in HTA, and 3.79–15.69% and 3.71–27.06% in RA, respectively. In total, 73.33% of road dust samples have more clay particles (≤2.5μm), while 66.7% of foliar samples have more silt particles (2.5–10μm). In addition, the maximum percentage of particles smaller than 10 μm was in IA for road dusts and AA for foliar dusts. The minimum percentage of particles smaller than 10 μm was in AA for road dusts and IA for foliar dusts. It was generally believed that the particulate matter produced by human sources was smaller than that produced by natural sources [26, 32]. The results showed the affection of human activity was stronger in IA than in AA.

D_10_, D_50_, D_90_, and D_av_ were basic parameters of particle size, which were chosen to examine the correlation analysis with specific area of particle (Table 1). After comparative analysis, D_av_ had the best correlation with specific area regardless of foliar versus road dust. The specific area was inversely proportional to D_av_, and the relative coefficients were -0.748 in foliar dusts and -0.723 in road dusts. Thus, there was a significant negative correlation between particle size and specific area. Consistent with the trend reported in the literature, the concentration of heavy metals decreased with the increasing of particle size [33, 34]. Smaller particles had a larger specific area that might absorb more contaminants.

### Distribution of heavy metals in foliar and road dusts

The statistical concentration data for 9 heavy metals in dusts with background soil values of Panzhihua were compared in Table 2. For foliar dusts, the maximum and minimum values of metals were 1.51 and 5.63mg/kg (Cd), 228.85 and 1504.7mg/kg (Cr), 113.39 and 220.48 mg/kg (Cu), 7.40×10^4^ and 2.06 ×10^5^mg/kg (Fe), 765.79 and 4718.07 mg/kg (Mn), 51.35 and 159.71 mg/kg (Ni), 130.74and 695.65 mg/kg(Pb), 445.51 and 1919.54 mg/kg (V),and 450.72 and 2010.85 mg/kg (Zn), respectively. In the case of road dusts, the maximum and minimum values of metals were 0.32 and 1.32mg/kg (Cd), 126.43 and 1221.04 mg/kg (Cr), 43.68 and 177.27 mg/kg (Cu), 3.50 × 10^4^ and 1.30 × 10^5^ mg/kg (Fe), 661.78 and 3906.82 mg/kg (Mn), 27.55 and 87.16 mg/kg (Ni), 32.48 and 264.91 mg/kg (Pb), 444.85 and 1781.69 mg/kg (V), and 176.04 and 706.52 (Zn), respectively.

**Table 2:** Concentrations of heavy metals in foliar and road dusts of Panzhihua (mg·kg^-1^).

Study Area	Sampling point	Cd		Cr		Cu		Fe		Mn		Ni		Pb		V		Zn	
		FD	RD	FD	RD	FD	RD	FD	RD	FD	RD	FD	RD	FD	RD	FD	RD	FD	RD
IA	S01	2.16	0.32	278.31	189.42	113.30	103.09	9.08×10^4^	6.54×10^4^	1537.95	1828.92	60.93	43.48	237.33	73.26	809.02	730.00	581.23	186.54
	S02	2.11	0.74	306.50	291.97	131.81	93.34	1.03×10^5^	9.58×10^4^	1595.21	2192.03	71.12	66.44	261.26	61.90	763.43	911.02	691.05	338.83
	S03	2.77	0.72	265.58	224.99	120.57	249.93	9.33×10^4^	1.11×10^5^	1533.34	2387.22	57.94	49.04	443.08	54.48	864.96	761.57	776.31	295.29
AA	S04	1.51	0.81	228.85	287.85	135.86	119.12	7.40×10^4^	1.59×10^5^	765.75	2573.84	44.61	76.25	327.56	67.56	445.51	895.61	450.72	436.84
	S05	2.87	0.50	1504.70	1221.04	124.22	57.79	2.06×10^5^	1.39×10^5^	2282.99	3906.82	159.71	87.16	436.50	108.93	1478.51	1781.69	2010.85	706.52
	S06	5.63	0.48	447.57	177.35	142.94	43.68	1.41×10^5^	3.50×10^4^	1740.20	661.78	94.51	55.41	695.65	32.83	927.30	240.81	1350.19	125.30
	S07	1.65	0.94	291.51	366.30	113.39	85.50	8.98×10^4^	1.21×10^5^	1306.52	2163.77	67.80	63.20	163.93	50.97	556.18	845.00	528.13	281.54
HTA	S08	2.75	0.81	303.25	254.49	170.23	97.50	1.06×10^5^	8.28×10^4^	1551.74	2100.20	78.10	53.49	344.77	57.33	667.96	717.76	742.45	276.99
	S09	2.95	0.93	362.45	188.88	184.53	83.46	1.31×10^5^	1.09×10^5^	1904.60	1838.86	78.88	45.76	423.36	49.93	1009.39	623.74	1024.28	451.00
	S10	3.24	0.86	531.92	294.42	220.48	177.27	1.44×10^5^	1.30×10^5^	2806.12	2038.32	84.24	53.78	372.72	264.91	1829.10	807.91	1087.77	408.89
	S11	2.44	0.86	493.61	190.89	173.85	46.00	1.08×10^5^	8.27×10^4^	2630.89	1438.69	65.05	33.07	222.55	72.09	1919.54	650.50	811.43	243.76
RA	S12	2.05	0.79	509.66	191.78	120.01	107.28	9.14×10^4^	1.01×10^5^	2552.84	1749.71	51.35	65.75	223.13	32.48	1877.00	637.06	550.97	251.84
	S13	2.01	1.21	478.11	284.07	175.50	171.42	9.56×10^4^	1.00×10^5^	2644.08	1705.95	59.45	53.97	157.66	141.03	1533.62	791.99	666.66	495.32
	S14	1.66	0.58	604.25	181.88	139.38	51.31	9.39×10^4^	1.27×10^5^	3479.68	1790.18	61.18	39.34	163.95	70.41	1517.85	670.31	544.97	232.69
	S15	4.64	1.32	878.58	126.43	134.48	63.48	1.17×10^5^	8.55×10^4^	4718.07	1386.96	72.32	27.55	130.74	48.55	1695.59	444.85	619.27	176.04
Mean value		2.70	0.79	498.99	298.12	146.70	103.34	1.12×10^5^	1.04×10^5^	2203.33	1984.22	73.81	54.25	306.95	79.11	1193.00	767.32	829.09	327.16
Standard deviation		0.96	0.23	283.85	231.92	30.67	50.72	2.85×10^4^	2.64×10^4^	869.50	600.25	23.53	14.44	130.33	50.37	461.32	281.77	354.12	128.54
Variation coefficient(%)		35.46	29.21	56.88	77.79	20.91	49.08	25.43	25.38	39.46	30.25	31.88	26.62	42.46	63.67	38.67	36.72	42.71	39.29
Soil background values		1.92		273.00		49.20		5.43×10^4^		1011.00		92.80		23.60		179.00		165.00	

More specifically, the maximum contents of Cr, Mn, Ni, V, and Zn in road dusts and Cr, Fe, Ni, and Zn in foliar dusts were concentrated in the S05 site. Though S05 was sited in AA, there were exist pollution sources around this site. The maximal values of Cu, Fe, and Pb in road dusts and Cu in foliar dusts were appeared in S10, which located in the busiest section of the traffic. The traffic jam and tail gas may be the pollution sources [30, 35]. It was to be observed that the contents of Cd, Cr, Cu, Ni, Pb, V, and Zn were higher in foliar dusts than in roads dust in almost all of the samples. Based on the results of previous section, foliar dusts had a smaller particle size than road dusts. Thus, the concentration of heavy metals was inversely proportional to the particle size, for the smaller particle size had a larger specific area and could absorb more substance, as previous studies had shown [11, 25, 33, 34]. However, the contents of Fe and Mn were nearly independent with particle size in 50% of the samples.

The average values of each heavy metal (Cd, Cr, Fe, Mn, Cu, Pb, V, and Zn) in foliar dusts were found to be substantially higher than their background soil values, and surpassing times were 0.41, 0.83, 1.98, 1.06, 1.18, 12.01, 5.66, and 4.02, respectively. Furthermore, the average contents of Cr, Fe, Mn, Cu, Pb, V, and Zn in road dusts were also 0.09, 1.10, 0.92, 0.96, 2.35, 3.29, and 0.98 times higher than those of background soil values, respectively. For all of the above elements, a partial anthropogenic contribution may be involved [30]. However, the concentration ranges of Cd in road dusts and Ni in foliar and road dusts were lower than corresponding background levels, showed the less anthropogenic influence to them.

In addition, 9 heavy metals had certain characteristics in different land-use districts of Panzhihua City. For road dusts, Cr, Fe, Mn, Ni, V, and Zn appeared to be more abundant in samples of AA (Table 3). Cr was predominant in IA. Pb in HTA was higher than that observed in other areas, which may be related to gasoline additives and combustion [16]. In the case of foliar dusts, Cd, Cr, Fe, Ni, Pb and Zn were relatively higher in AA. The highest contents of Mn and V were in RA. In HTA, Cu is slightly higher than other areas. For the relative high concentration of heavy metals in both foliar and road dusts of AA, further investigation was adopted. The results show there were two under-developed tile kilns in this area between S05 and S06. The ash was discharged without any treatment and returned to ground surface through atmospheric deposition, and waste residue was banked up in the field, which had high contents of Cr, Fe, Ni, Pb, V, Zn, and so on [15, 24]. In addition, the AA was at the downwind direction of IA, the atmospheric dusts from the latter might influence the former.

**Table 3:** Mean concentration of heavy metals in dusts of Panzhihua and other selected cities(mg·kg^-1^).

Samples	Country	City	Land use	Cd	Cr	Cu	Fe	Mn	Ni	Pb	V	Zn
RD	China	Panzhihua (This study)	IA	0.59	235.46	148.79	90737.50	2136.06	52.99	63.21	800.86	273.55
			AA	0.68	513.14	76.52	113598.78	2326.55	70.51	65.07	940.78	387.55
			HTA	0.87	232.17	101.06	101180.23	1854.02	46.53	111.07	699.98	345.16
			RA	0.98	196.04	98.37	103505.78	1658.20	46.65	73.12	636.05	288.97
		Urumqi[36]		1.97	186.00	179.00			289.70	187.00		227.00
	Australia[37]	Clearview Estate	Residential area	0.51	14.80	131.40	7220.00	200.00	7.92	32.50		296.60
		Nerang	Industrial area	0.19	3.96	65.50	4230.00	90.00	6.11	25.70		176.40
		Benowa	Residential and Commercial area	0.35	9.37	98.40	5730.00	150.00	7.01	29.10		236.50
		Surfers Paradise	Commercial area	0.54	3.16	70.80	2980.00	60.00	4.53	38.40		90.40
	Italy	Gela[38]	Peripheral area		20.00	49.00		370.00	29.00	69.00	55.00	218.00
			Industrial area		38.00	48.00		400.00	38.00	35.00	90.00	196.00
			Urban area		43.00	104.00		380.00	36.00	72.00	64.00	220.00
	Spain	Murcia Region[6]	High density population	1.07	39.30	134.00			41.70	117.00		203.00
			Medium density population	1.55	28.70	75.40			37.10	85.70		149.00
			Low density population	1.28	23.00	68.70			38.40	75.30		105.00
			Natural area (no population)	1.19	20.40	19.50			33.00	27.80		50.30
FD	China	Panzhihua (This study)	IA	2.35	283.46	121.89	95622.92	1555.50	63.33	313.89	812.47	682.86
			AA	2.92	618.16	129.10	127653.15	1523.87	91.66	405.91	851.88	1084.97
			HTA	2.85	422.81	187.27	122430.85	2223.34	76.57	340.85	1356.50	916.48
			RA	2.59	617.65	142.34	99406.10	3348.67	61.08	168.87	1656.02	595.47
		Huizhou city[5]	Commercial and traffic type	7.80	241.50	324.80				512.00		1782.40
			Electronic Industry area	8.40	237.00	1313.00				462.00		1860.00
			Power station	12.80	927.60	914.60				184.00		204.60
			Residential area	7.40	215.60	235.20				460.00		1223.00
			Park	6.20	202.00	228.80				434.00		1127.00
	Austria	Vienna[21]	Urban area	n.d.		30.00	2136.00	n.d.		18.00		311.00
			Suburban area	n.d.		24.70	1164.00	n.d.		13.60		285.00
			Rural area	n.d.		20.10	7.50	n.d.		5.80		203.00

n.d.: Not detected.

Although there are no universally accepted sampling and analytical procedures for geochemical studies of urban deposits, it is common practice to compare mean concentrations of heavy metals in dusts [15, 16]. In Table 3, the mean concentrations of Cd, Cr, Cu, Fe, Mn, Ni, Pb, V, and Zn measured in foliar and road dusts in different land-use districts were compared with data reported for other cities. The results showed average values of Cr, Fe, Mn, V, and Zn in road dusts sampled in Panzhihua (this work) were higher than all of the other cities in the world with which they were compared. The mean values of Cd, Cu, Ni, and Pb in road dusts from Urumqi (China) were the highest [36], and their contents in Panzhihua City were similar to those sampled in cities of Australia [37], Italy [38] and Spain [6]. In Murcia Region of Spain, there were obvious trends for the metal contents except Cd increased with the increasing density of the population. However, in Panzhihua or in the cities of Australia and Italy, the heavy metal contents were not obviously higher in IA than RA or AA, as expected.

There were not abundant data for foliar dusts in previous study, so only three cities were compared in the following. In Panzhihua, the heavy metal concentrations in foliar dust were relatively higher in AA. One reason was the sampling sites of AA were at the downwind of IA. And the second reason was the tile kilns in AA. Both of the two reasons might cause the excessive deposition of heavy metals. However in another city of China (Huizhou City) [5], they were higher in commercial and industrial area. In the city of Vienna in Austria [21], there was a lower content of heavy metals in rural area than urban area. In total, the heavy metal concentrations in Vienna were the lowest when compared to the other two cities. The contents of Cd, Cu, and Zn in Panzhihua City were lower than Huizhou City, and Pb was similar with the latter. Cr was higher than Huizhou City except in its power station area.

### Identification of heavy metal sources

#### Cluster analysis

**Fig 4:**
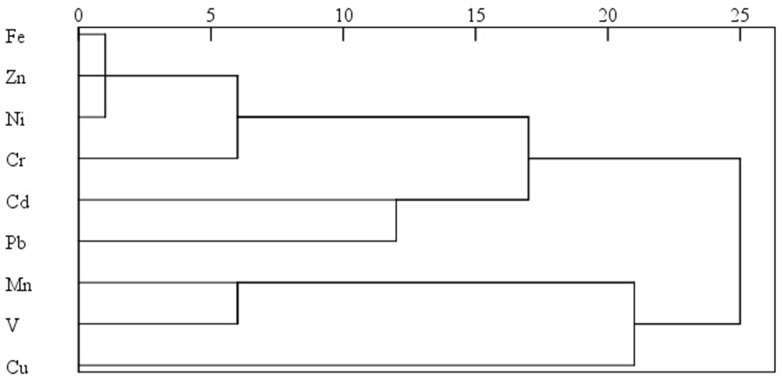
Dendogram of heavy metal concentrations in foliar dusts of Panzhihua.

**Fig 5:**
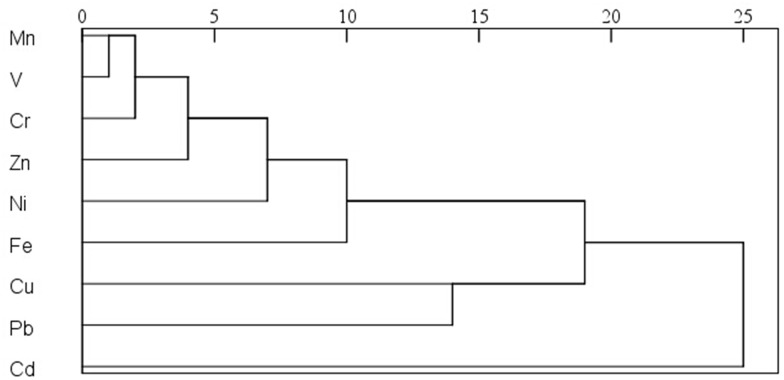
Dendogram of heavy metal concentrations in road dusts of Panzhihua.

The dendrograms calculated by cluster analysis showed the similarities of heavy metals in foliar and road dusts of Panzhihua (Figs 4 and 5). The x-axis represented the similarity between variables, and a higher correlation degree was associated with a smaller x-axis value. The results for the contaminants studied indicated four clusters in foliar dusts: (1) Fe-Zn-Ni-Cr; (2) Cd-Pb; (3) Mn-V; and (4) Cu in terms of similarities. However, cluster 1 was moderately associated with cluster 2, leading to a main cluster named A in Fig 4. Cluster 3 was associated with cluster 4, leading to a main cluster named B. In addition, metals in road dusts could be divided into four clusters: (1) Mn-V-Cr-Zn-Ni-Fe; (2) Cu; (3)Pb; and (4) Cd. The cluster 1 was moderately associated with cluster 2 and 3, leading to a main cluster named C in Fig 5. The findings indicated that contaminants in foliar dusts, such as cluster A (Fe, Zn, Ni, Cr, Cd, Pb) and cluster B (Mn, V, Cu) were partly derived from each other and might have some common anthropogenic sources. For road dusts, cluster C (Fe, Zn, Ni, Cr, Pb, Mn, V, and Cu) might have similar anthropogenic sources, while Cd appeared to be originated from unique sources.

#### Enrichment factor analyses

**Fig 6:**
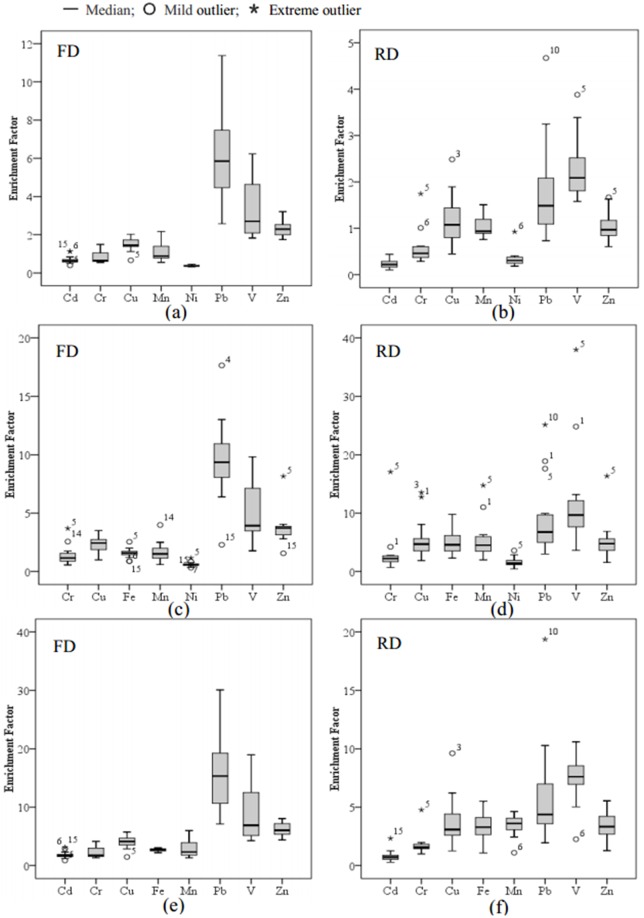
Boxplot of EFs for heavy metals in foliar and road dust samples of Panzhihua. (a) and (b): Fe was used as reference element; (c) and (d): Cd was used as reference element; (e) and (f): Ni was used as reference element. FD: Foliar Dust; RD: Road Dust.

Enrichment factor method reflects the degree of heavy metal pollution in one region according to the change in ratios between samples and background values, which is used to judge the anthropogenic sources and pollution degree. Fe, Al, and Li are frequently chosen as reference metals [16]. Alternatively, one could use any other metal believed to have no or minimal anthropogenic origin and stable chemical properties that is minimally influenced by other metals and widely distributed in the crust [15, 16]. The former results of cluster analysis indicated the sources of Cd in road dusts and Cu in foliar dusts differed from the other elements. In addition, only the contents of Cd and Ni were lower than the background soil. Fe was frequently used as a reference metal, while its concentrations were higher than the background value of soil and positively correlated with many metals in this study. In order to compare which elements was the most suitable, the EFs were calculated based on Fe, Cd and Ni as reference metals (Fig 6).

With Fe as the reference metal, the general pollution degree was low (Fig 6a and 6b). Only EFs of Pb, V, and Zn were higher than 2 in foliar dusts, and partly Pb and V in road dusts were moderate in enrichment. Metals with Enrichment factor> 10 were always believed to derive from human activities [16]. Therefore, the main sources of metal were natural with Fe as the reference metal, which needs to be certified in the following analysis.

When Cd was chosen as a reference metal, the heavy metals most likely to cause risk in Panzhihua dust samples were in the order of Pb (significant enrichment) >V (moderate and significant enrichment) >Zn (moderate enrichment) >Cu, Cr, Fe, and Mn (minimal and moderate enrichment), and >Ni (minimal enrichment) forfoliar dusts. Due toroad dusts, the order was as follows: V (significant enrichment) >Pb, Zn, Cu, Fe, and Mn (moderate and significant enrichment), and > Cr, Ni (minimal and moderate enrichment). However, there were too many outliers as showed in Fig 6c and 6d.

With Ni as reference metal, part of the EFs of Pb and V were close to or higher than 10 and even 20 (Fig 6e and 6f), which showed that they were significantly enriched in the dusts of Panzhihua and mainly originate from anthropogenic sources. Zn were moderate and showed significant enrichment; Cu, Cr, Fe, and Mn showed moderate enrichment; and Cd showed minimal and moderate enrichment, which were mainly from natural sources. Although the orders of enrichment levels of metals vary depending on as the choice of reference metal, it was clear that the enrichments of Pb and V were quite high in all cases. The enrichment levels of metals were similar when the reference value was Cd and Ni, while it was too low to reflect the real metal pollution with Fe as reference metal. While, the existence of so many outliers of EFs with Cd as reference value may because of its lower distribution in crust and instability in environment.

Therefore, Ni was the best choice as reference value in this work and showed no or minimal anthropogenic influence, which was very important. Overall, Panzhihua road and foliar dusts were highly contaminated with Pb and V. The extreme outlier EFs of Pb in road dusts was 19.5 at S10, where have the maximum vehicle flow. Tail gas may be the most important sources of Pb. The extreme outlier EFs of Cd and Cr were at the S15 and S5 site respectively. For all of them were lower than 10, they were very little affected by human activities. Although Zn was derived mostly from the same source as Cr, Cu, Fe and Mn, it has enriched to relatively higher levels in foliar dusts, indicating its emissions were more than the other metals. There was no pollution of Cd in the study area.

#### Principal component analysis

Principal component analysis was performed for identifying the probable metal sources from dusts of Panzhihua City (Tables 4–7). As expected, four principal factors of foliar dusts were acquired (Table 4), and the principal components were shown in Table 5. Ni, Zn, Fe, and Cr constituted the first column with factors > 0.82, and they were mainly derived from common sources. The second column contained Mn and V, indicating that they originated from the same origins. Cd and Pb appeared in the third column, so Cd in foliar dusts might have other anthropogenic sources besides natural sources. Finally, Cu with a factor of 0.97 was in the fourth column with its sources differing from the other materials. Meanwhile, there were five principal factors for road dusts (Table 6). The first column contained Cr, V, Mn, Zn, and Ni, which might be derived from common sources (Table 7). The other four columns were Fe, Cd, Pb, and Cu, and each of them arose from different origins.

**Table 4:** Eigenvalues and variance ratios of metals in foliar dusts of Panzhihua.

Principal component	Initial eigenvalues			Extracted eigenvalues			Rotated eigenvalues		
	Eigenvalues	% of variance explained	% of cumulative	Eigenvalues	% of variance explained	% of cumulative	Eigenvalues	% of variance explained	% of cumulative
1	4.33	48.07	48.07	4.33	48.07	48.07	3.66	40.65	40.65
2	2.26	25.07	73.14	2.26	25.07	73.14	2.31	25.63	66.27
3	1.24	13.74	86.88	1.24	13.74	86.88	1.53	17.00	83.27
4	0.81	9.05	95.92	0.81	9.05	95.92	1.14	12.65	95.92
5	0.26	2.91	98.83						
6	0.06	0.68	99.51						
7	0.03	0.32	99.83						
8	0.01	0.09	99.93						
9	0.01	0.07	100.00						

**Table 5:** Rotated component matrix for data of metals in foliar dusts of Panzhihua.

Metals	Percentage			
	PC1	PC 2	PC 3	PC 4
Ni	0.97			
Zn	0.94			
Fe	0.94			
Cr	0.82			
Mn		0.97		
V		0.86		
Cd			0.94	
Pb			0.67	
Cu				0.97

**Table 6:** Eigenvalues and variance ratios of metals in road dusts.

Principal component	Initial eigenvalues			Extracted eigenvalues			Rotated eigenvalues		
	Eigenvalues	% of variance explained	% of cumulative	Eigenvalues	% of variance explained	% of cumulative	Eigenvalues	% of variance explained	% of cumulative
1	4.75	52.82	52.82	4.75	52.82	52.82	3.94	43.82	43.82
2	1.40	15.53	68.35	1.40	15.53	68.35	1.18	13.08	56.90
3	1.09	12.06	80.41	1.09	12.06	80.41	1.15	12.76	69.66
4	0.76	8.44	88.85	0.76	8.44	88.85	1.12	12.40	82.06
5	0.45	4.98	93.83	0.45	4.98	93.83	1.06	11.76	93.83
6	0.34	3.78	97.61						
7	0.13	1.47	99.07						
8	0.07	0.76	99.83						
9	0.02	0.17	100.00						

**Table 7:** Rotated component matrix for data of metals in road dusts of Panzhihua.

Metals	Percentage				
	PC1	PC 2	PC 3	PC 4	PC1
Cr	0.96				
V	0.94				
Mn	0.87				
Zn	0.84				
Ni	0.68				
Fe		0.83			
Cd			0.97		
Pb				0.97	
Cu					0.99

#### Potential source identification

Cluster analysis is only used to show the correlation between sources of different metals. The enrichment factor method is used to explain the anthropogenic or natural sources, while it cannot give more specific information. Principal component analysis is widely used to identify possible sources of heavy metals, while it cannot illustrate whether the pollution comes from natural sources or anthropogenic sources and the correlation between each other. Thus all of the statistical data including cluster, enrichment factor and principal component analysis were compared to identify probable sources of heavy metals in dusts of Panzhihua. The main classifications of heavy metals in dust samples by principal component analysis and cluster analysis were almost the same. There were few differences between the principal components of foliar and road dusts, so they have similar sources.

The high EFs of Pb suggested it was the primary pollution element in the study area. And anthropogenic activities were its major sources. While the highest concentration of Pb was not in HTA, as expected, but was almost in AA. So Pb might mainly originate from the waste incineration (straw burning) and lead-fuel combustion [10, 39, 40].

V was another contaminant in the dusts of Panzhihua. For Panzhihua is the most famous base of V-Ti magnetite production in the world, so V content in soil is higher than the global vanadium background value. Nevertheless, the V concentrations in dusts also were higher than its background soil values throughout the study area. The high concentration and EFs of V indicated it mainly originated from anthropogenic origins, such as the combustion of fossil fuels, mining of vanadium-titanium-magnetite, steel-iron refining, electronics and dyeing, etc.[41–43]. Mn had a significant positive correlation with V, and therefore, they had common sources.

According to the results of cluster analysis and principal component analysis, Zn, Ni, Cr, and Fe were in the same or similar groups. When the significance level was p<0.01, there was a significant positive correlation with each other. Thus, they were from the same type of sources. Because their EFs were between 2 and 7, there was moderate pollution of Zn, Ni, Cr and F, whose main sources were anthropogenic. Meanwhile, they also had a positive correlation with V and Mn, though they were not in the same clusters in foliar dusts. Thus, Zn, Ni, Cr, and Fe were derived mostly from the same sources as V and Mn such as metal smelting and coal combustion. Furthermore, parts of them originated from fugitive dust and traffic activities [30, 44]. Ni was slightly different from the other metals based on its lower content than the background value of soil. Though it was minimally enriched in both foliar and road dusts, it was mainly from human activities.

Cu was moderately polluted, and Cd was minimally enriched in Panzhihua. Because none of metals were strongly correlated with Cd in road dusts and Cu in all dust samples, they might have different sources from other metals. The probable origins of Cu might be corrosion of alloys used in vehicle components, vehicle covers, or other metallic surfaces and materials, as previous references had shown [15, 30, 39, 44, 45]. And Cd may originated from different industrial or traffic emissions.

## Conclusion

The present study demonstrated the distribution and sources of heavy metals (V, Pb, Cd, Cu, Zn, Ni, Cr, Fe, and Mn) in road and foliar dusts of Panzhihua City. Most of the metals in dust samples were higher than soil background values of Panzhihua except Ni. Foliar dust samples had a smaller particle size and higher heavy metal contents than road dusts. The results of enrichment factor analyses showed Pb and V were the main pollutants; Zn, Cr, Cu, Fe, and Mn were moderately contaminated; and Cd and Ni were minimally enriched in foliar and road dusts.

Pb, as the primary pollution element in the region, probably originated from field burning and lead-fuel combustion. As a famous location of V-Ti magnetite production globally, V was another main contaminant, and its main sources were industry activities such as combustion of fossil fuels, mining of vanadium-titanium-magnetite, steel-iron refining, electronics and dyeing, etc. The main sources of Zn, Ni, Cr, Fe, and Mn were partly the same as V, which also originated from fugitive dust and traffic activities. None of the metals were strongly correlated with Cd in road dusts and Cu in all dust samples, and therefore, they had different sources from others. Potential origins of Cu were corrosion of alloys used in vehicle components, vehicle covers, or other metallic surfaces and materials. Industrial or traffic emissions different from other heavy metals were the main sources of Cd. The heavy metal contents of foliar and road dusts were relatively higher in agricultural area than other areas, which were due to the influences of ash from tile kilns around it. Environments of agricultural and residential area were closely related with health of local residents, so more attention should be paid to heavy metal contamination in such area, especially Pb and V.
